# A Randomized Controlled Trial Comparing Isobaric Versus Hypobaric Plus Isobaric Bupivacaine in Thoracic Segmental Spinal Anesthesia for the Reduction of Shoulder Pain During Laparoscopic Cholecystectomy

**DOI:** 10.7759/cureus.97048

**Published:** 2025-11-17

**Authors:** Richa Chandra, Imran Ahmed Khan, Naresh W Paliwal, Sohel Anjum, Anmol Singh

**Affiliations:** 1 Anaesthesiology, Rohilkhand Medical College and Hospital, Bareilly, IND; 2 Community Medicine, KMC Medical College and Hospital, Maharajganj, IND; 3 Anaesthesiology, Dr. Panjabrao Deshmukh Memorial Medical College, Amravati, IND; 4 Anaesthesiology, Glenfield Malla Reddy Brain Heart Hospital, Hyderabad, IND; 5 Anaesthesiology, Adesh Institute of Medical Sciences and Research, Bathinda, IND

**Keywords:** bupivacaine, laparoscopic cholecystectomy, patient satisfaction, postoperative complications, regional anesthesia, shoulder pain

## Abstract

Introduction

Laparoscopic cholecystectomy (LC) is traditionally performed under general anesthesia (GA). However, thoracic segmental spinal anesthesia (TSSA), where low doses of local anesthetics (LA), often with adjuvants, are used at thoracic spinal levels, is also being explored by some researchers. Shoulder pain is a common issue during LC, adversely impacting the patient’s perioperative experience. A combination of hypobaric and isobaric LA at the thoracic level has been described to mitigate this complication. The primary objective of the study was to compare the efficacy of a combination of hypobaric and isobaric bupivacaine versus isobaric bupivacaine alone during TSSA in LC in reducing intraoperative shoulder pain. The secondary objectives were to assess the incidence of adverse effects (hypotension, bradycardia, nausea, vomiting, etc.) and to evaluate patient and surgeon satisfaction.

Methods

This randomized, controlled, open-label study was conducted at a tertiary care center after receiving ethical approval and registration at the Clinical Trial Registry of India. A total of 90 patients were recruited, with 45 participants in each group, aged 20-70 years, with ASA Physical Status I-II, scheduled for elective LC. Exclusion criteria included BMI > 35, contraindications to regional anesthesia, allergy to study drugs, spinal deformity, and previous abdominal surgery. Patients were randomly assigned to two groups using computer-generated random numbers. Anesthesia was provided by a senior consultant proficient in TSSA. Group 2 received hypobaric and isobaric bupivacaine, and Group 1 received only isobaric bupivacaine. Both groups received 11 mg bupivacaine with 5 μg dexmedetomidine in TSSA. Data were collected using Microsoft Excel (Microsoft® Corp., Redmond, WA, USA) and analyzed using IBM SPSS Statistics for Windows, Version 23 (Released 2015; IBM Corp., Armonk, NY, USA). Continuous variables were expressed as means ± SD and analyzed using an independent t-test or the Mann-Whitney U-test, depending on the distribution of data. Categorical variables were compared using the Chi-square or Fisher’s exact test. A p-value of <0.05 was considered statistically significant.

Results

All 90 patients in both groups successfully underwent LC under TSSA with no conversion to GA. The mean age of the participants was 49.11 ± 7.4 years with 58 (64.4%) females. Both groups were comparable in terms of demographic parameters. Intraoperative clinical parameters were comparable in both groups, without any statistically significant differences. Six (13.3%) patients in Group 1 and five (11.1%) patients in Group 2 had hypotension, which was easily corrected with a fluid bolus and a single 6 mg dose of intravenous mephentermine. Six (13.3%) patients in Group 1 reported shoulder pain, whereas in Group 2 only one (2.2%) patient had shoulder pain intraoperatively. Patient and surgeon satisfaction scores were better in Group 2, which was statistically significant. The number needed to treat (NNT) of nine indicates that approximately nine patients would need to receive the hypobaric + isobaric regimen to prevent one case of intraoperative shoulder pain.

Conclusions

LC can be performed successfully under TSSA with stable hemodynamics. The addition of hypobaric bupivacaine to isobaric bupivacaine provided better shoulder-tip pain control and fewer postoperative complications. Further studies with larger sample sizes are needed to validate these findings.

## Introduction

Laparoscopic cholecystectomy (LC) is a common surgical procedure performed to manage gallstone disease. Although laparoscopic procedures offer significant benefits, such as minimal scarring and faster recovery, complications like shoulder pain can adversely impact the patient’s perioperative experience. LC is traditionally performed under general anesthesia (GA). However, spinal anesthesia (SA), including lumbar spinal anesthesia (LSA) and thoracic segmental spinal anesthesia (TSSA), is increasingly being explored as an alternative anesthetic approach when GA carries considerable risk [1-3]. In TSSA, low doses of local anesthetics (LA), often with adjuvants, are used at thoracic spinal levels [4]. One of the notable complications associated with LC under SA is intraoperative shoulder pain, which can significantly affect patient comfort and recovery. This is often attributed to overstretching of the peritoneum and diaphragmatic irritation by carbon dioxide (CO₂) insufflation [5,6]. Shoulder pain during LC was reported in 25% of participants in a systematic review and meta-analysis conducted by Longo et al. [7], suggesting that shoulder pain is a common issue during LC. Another study reported shoulder-tip pain in 16.7% of participants using an isobaric drug [8]. To further explore this complication, a combination of hypobaric and isobaric LA at the thoracic level was described [9]. Hypobaric solutions spread cephalad against gravity in the sitting position [10]. We hypothesized that blocking phrenic nerve afferents with low-dose bupivacaine could mitigate shoulder pain. In this study, the aim of using low-concentration hypobaric bupivacaine was to provide a sensory cervical spread only, sparing the motor effect on the phrenic nerve and avoiding any respiratory compromise.

The primary objective of the study was to compare the efficacy of a combination of hypobaric and isobaric bupivacaine versus isobaric bupivacaine alone during TSSA in mitigating intraoperative shoulder pain in LC. The secondary objectives were to assess the incidence of perioperative adverse effects (e.g., hypotension and bradycardia) and postoperative nausea and vomiting (POVN). Patient and surgeon satisfaction were also assessed.

## Materials and methods

Study design and setting

This randomized, controlled, open-label study was conducted at a tertiary care center from December 2024 to January 2025 (two months) after receiving ethical approval from the Rohilkhand Medical College Ethical and Research Committee (vide approval letter number IEC/RMCH/011/2024/JUL, dated July 30, 2024). The study was prospectively registered with the Clinical Trial Registry of India (CTRI registration number CTRI/2024/11/076271, dated November 5, 2024; accessible at www.ctri.nic.in). Patient confidentiality was ensured, with data stored securely and accessible only to authorized personnel. Participants were informed about the risks and benefits of the anesthesia techniques, and written informed consent was obtained from all participants. The study adhered to the principles of the Declaration of Helsinki and the Good Clinical Practice guidelines.

Sample size calculation

A sample size of 40 patients per group would provide 80% power to detect significant differences at a 5% significance level (α = 0.05). A total of 90 patients were recruited, with 45 participants in each group to adjust for any loss to follow-up or conversion to GA.

Patient selection and randomization

**Figure 1:**
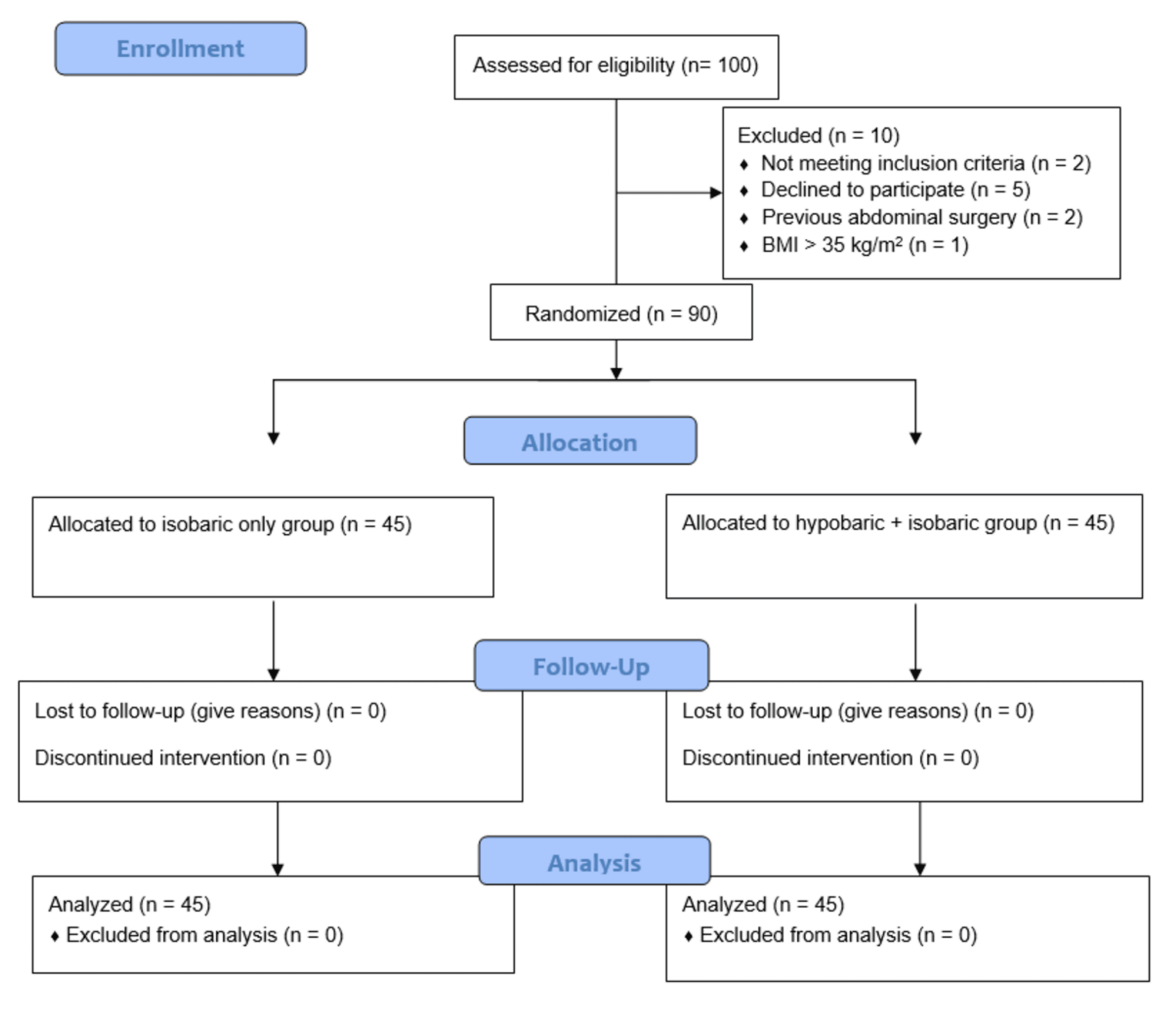
CONSORT flow diagram CONSORT, Consolidated Standards of Reporting Trials

We included patients aged 20 to 70 years with ASA Physical Status I and II, scheduled for elective LC. Exclusion criteria included BMI >35; contraindications to regional anesthesia (e.g., local infection and coagulopathy); allergy to study drugs; and spinal abnormalities (e.g., kyphosis and scoliosis). We also excluded patients with previous abdominal surgeries, a history of pancreatitis, acute cholecystitis, or empyema. Patients were randomly assigned to two groups (45 per group) using computer-generated random numbers. For allocation concealment, the serially numbered opaque sealed envelope method was used. Figure 1 shows the Consolidated Standards of Reporting Trials (CONSORT) flow diagram.

Study groups

For Group 1, TSSA was performed at T9-T10/T10-T11 with 2.2 mL of isobaric bupivacaine 0.5% and 5 μg dexmedetomidine. For Group 2, TSSA was performed at T9-T10/T10-T11 with 2 mL of hypobaric bupivacaine 0.1%, followed by 1.8 mL of isobaric bupivacaine 0.5% and 5 μg dexmedetomidine, administered after 45 seconds. Hypobaric bupivacaine 0.1% was freshly prepared by mixing 1 mL of 0.5% bupivacaine with 4 mL of sterile distilled water [10]. Both groups received similar amounts of bupivacaine (total dose = 11 mg) with dexmedetomidine.

Preoperative preparation and anesthesia procedure

Standard preoperative evaluation was done, including a complete clinical examination, complete blood count, renal function tests, liver function tests, electrocardiography (ECG) and, where indicated, chest X-ray, echocardiography, and subspecialty consultation.

Anesthesia was provided by a senior consultant proficient in TSSA. Patients were positioned sitting for the TSSA, and standard monitoring - including ECG, non-invasive blood pressure (NIBP), pulse oximetry (SpO₂), end-tidal carbon dioxide (EtCO₂), and a temperature probe - was applied, and their functions were verified. An intravenous cannula (20 or 18 G) was placed, and 10 mL/kg of Ringer's lactate was administered as preloading. Under aseptic precautions, TSSA was performed using a 27-G Quincke Babcock needle. For TSSA, the T9-T10/T10-T11 space was identified using anatomical landmarks (C7 spine, T7 scapular level, and L1, corresponding to the 12th rib). Before injection, a baseline sensory test (pinprick and cold) was performed at T10 (umbilicus), T4 (nipple), and C3-C5 (supraclavicular/shoulder) to confirm no sensory changes. Following the TSSA, patients were placed in the supine position with a 10-degree head-up tilt. Sensory blockade was assessed bilaterally using a multimodal approach: a pinprick test with a blunt sterile needle (assessing sharpness vs. dullness) and a cold sensation test with an ice swab (assessing cold vs. touch/warmth), starting at T10, progressing cephalad to T4 along the mid-clavicular line, and extending to C3-C5 if T4 was blocked, at one-minute intervals. The time of drug administration completion was considered “0 minutes.”

Motor blockade was assessed using the Bromage scale in the lower limb, and the Epidural Scoring Scale for Arm Movements (ESSAM) in the upper limb [11,12]. Time for the block to reach the T4 dermatomal level, and time to regression of the sensory and motor block, were also recorded. The overall duration of anesthesia was defined as the period (minutes) included between the onset and the complete regression of the sensory block. Block adequacy required T3-T4 to T12-L1 coverage for surgery, with C3-C5 in Group 2 for shoulder pain control. All the cases were performed by a senior surgeon, having vast experience with laparoscopic cases. The surgeon and the outcome assessor were blinded to the group of patients. Surgery was performed using four traditional ports, keeping the intra-abdominal pressure (IAP) at 10-12 mmHg.

Oxygen was administered at 4 L/min with nasal prongs. Vital signs were recorded every minute for the first 15 minutes, then every five minutes until the completion of surgery. Hypotension (systolic blood pressure, or SBP <90 mmHg) was treated with a fluid bolus and intravenous mephentermine (6 mg), and bradycardia (heart rate, or HR <50/min) with atropine (0.6 mg). Sedation was achieved with intravenous midazolam (1 mg) and fentanyl (1-1.5 μg/kg). Intraoperative shoulder pain was treated with tramadol (50 mg) and additional sedation, if needed, and intraoperative nausea and vomiting with ondansetron (4 mg). Shoulder pain was assessed using an 11-point numerical rating scale (NRS), recorded every five minutes during pneumoperitoneum, where 0 = no pain and 10 = worst pain. This scale is widely validated in postoperative and procedural pain assessment [13]. NRS ≥ 4 at any time during the operation was considered shoulder pain. The modified Ramsay’s sedation scale (0 = alert, 1 = easily arousable, 2 = awake with tactile stimulation, 3 = awake after verbal stimuli, and 4 = not arousable) was used to assess sedation [14,15]. Respiratory depression was defined as a respiratory rate (RR) of less than 10 breaths/min, or oxygen saturation of less than 90% by pulse oximetry, lasting at least three minutes.

Intraoperative incidents, like shoulder or abdominal pain, nausea, hypotension, and bradycardia, were recorded. Incidence of clinically significant shoulder pain (NRS ≥4) at any time during the intraoperative period was designated as shoulder pain present. Evacuation of intra-abdominal CO₂ was done as a routine in all the cases. The port site was infiltrated with 0.25% bupivacaine. Postoperatively, as described above, pain was assessed using NRS. All patients received paracetamol 1 g and diclofenac 75 mg intravenously, 12-hourly for one day, to manage postoperative pain.

All patients were asked to assess their degree of satisfaction with the procedure when they came for suture removal, at 8-10 days postoperatively. Patient and surgeon satisfaction was assessed using a 10-point Likert scale (1-10), which comprised 1 for "very dissatisfied" and 10 for "very satisfied."

Statistical analysis

Data was collected using Microsoft Excel (Microsoft® Corp., Redmond, WA, USA). Data were analyzed using IBM SPSS Statistics for Windows, Version 23 (Released 2015; IBM Corp., Armonk, NY, USA). Continuous variables (e.g., age, block duration, and satisfaction score) were expressed as means ± SD, and analyzed using an independent (unpaired) t-test or Mann-Whitney U-test, depending on the distribution of the data. Categorical variables (e.g., incidence of shoulder pain) were compared using the Chi-square or Fisher’s exact test. A p-value of <0.05 was considered statistically significant.

## Results

The eligibility of 100 patients to participate in the study was assessed. The study excluded 10 subjects: five declined to participate, and five failed to fulfill the inclusion criteria. A total of 90 patients were included in this study, with 45 participants in each group. All patients in both groups successfully underwent LC under TSSA, with no conversion to GA. The mean age of the participants was 49.11 ± 7.4 years, with 58 (64.4%) females (Table 1). Both groups were comparable in terms of demographic parameters.

**Table 1:** Demographic and clinical characteristics of the participants ^a^ t-value (Independent samples t-test); ^b^ Chi-square value (Chi-square test) SD, standard deviation; BMI, body mass index; ASA-PS, American Society of Anesthesiologists-Physical Status

		Group 1 (n = 45)	Group 2 (n = 45)	Test-statistic	p-value
Age (mean ± SD)		48.53 ± 7.4	49.69 ± 7.33	-0.74^a^	0.46
Gender (Number (%))	Male	14 (43.8)	18 (56.2)	0.78^b^	0.34
	Female	31 (53.4)	27 (46.6)		
Height (mean ± SD)		157.47 ± 2.9	156.76 ± 2.9	0.12^a^	0.25
Weight (mean ± SD)		56.69 ± 3.2	56.27 ± 3.3	0.73^a^	0.54
BMI (mean ± SD)		22.86 ± 1.0	22.9 ± 1.3	1.3^a^	0.86
ASA-PS (Number (%))	I	39 (54.2)	33 (45.8)	2.5^b^	0.11
	II	6 (33.3)	12 (66.7)		

Anesthesia characteristics of the participants are provided in Table 2. The duration of the sensory blockade was longer in Group 2 (162 ± 20 minutes) than in Group 1 (145 ± 18 minutes). Eleven patients in Group 1, and 10 patients in Group 2, had complete motor blockade in the lower limbs, and none had motor blockade in the upper limbs. The upper sensory level achieved in Group 1 was T3-4, and in Group 2, it was up to C4-5. Elbow flexion, wrist flexion, and finger movements were intact in all patients.

**Table 2:** Anesthesia characteristics of the participants ^a ^t-value (Independent samples t-test); ^b ^Chi-square value (Chi-square test) Patient and surgeon satisfaction was assessed using a 10-point Likert scale (1-10), which comprised 1 for "very dissatisfied" and 10 for "very satisfied." SD, standard deviation

Anesthesia characteristics		Group 1 (n = 45)	Group 2 (n = 45)	Test-statistic	p-value
Duration of the sensory blockade (minutes), mean ± SD		145 ± 18	162 ± 20	-4.24^a^	0.001
Max Bromage scale	1	26	30	1.1^b^	0.77
	2	4	3		
	3	4	2		
	4	11	10		
Duration of the motor blockade (minutes), mean ± SD		122 ± 15	135 ± 16	-3.93^a^	0.002
Time for first postoperative analgesia (minutes), mean ± SD		178 ± 24	200 ± 26	-4.17^a^	<0.001
Patient satisfaction score, mean ± SD		8.7 ± 0.8	9.2 ± 0.6	-3.36^a^	0.003
Surgeon satisfaction score, mean ± SD		8.5 ± 0.9	9.0 ± 0.7	-2.94^a^	0.005

**Figure 2:**
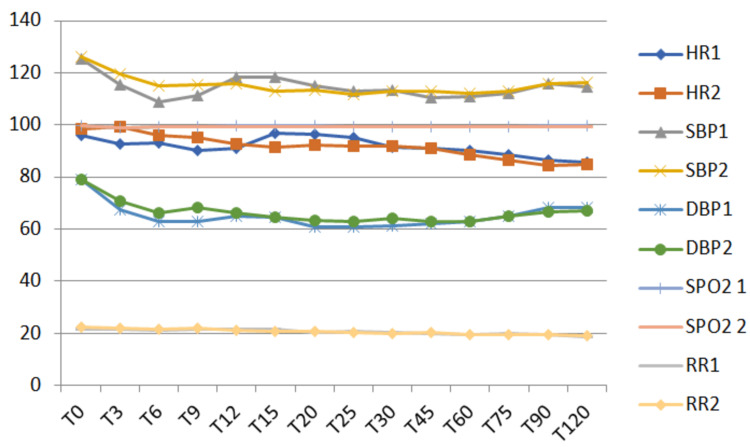
Clinical parameters of the participants Here, the x-axis denotes time and the y-axis different clinical parameters. T, time (minutes); T0, baseline; HR, heart rate (beats/minute); SBP, systolic blood pressure (millimeter mercury); DBP, diastolic blood pressure (millimeter mercury); SpO₂, oxygen saturation (percentage); RR, respiratory rate (number/minute)

Clinical parameters, in terms of HR, RR, NIBP, and SpO₂, were comparable in both groups, without any statistical difference, as shown in Figure 2. The Ramsay scale was found to be 1-2 in all cases throughout the procedure. Patient and surgeon satisfaction scores were better in Group 2, which was statistically significant.

Table 3 shows perioperative complications of the participants. The incidence of intraoperative shoulder pain was 2.2% (1/45) in the hypobaric + isobaric group, versus 13.3% (26/45) in the isobaric-alone group (p = 0.11). Six patients in Group 1, and five patients in Group 2, had hypotension, which was easily corrected with a fluid bolus and a single 6 mg dose of intravenous mephentermine. Six (13.3%) patients reported shoulder pain in Group 1, and in Group 2, only one (2.2%) patient had shoulder pain intraoperatively. The absolute risk reduction (ARR) for shoulder pain with the hypobaric + isobaric combination was 11.1% (95% CI: 0.29% to 21.93%), yielding a number needed to treat (NNT) of nine (95% CI: 5-345). The requirement of intraoperative analgesics was higher (5, 11.1%) in Group 1 than in Group 2 (1, 2.2%). One patient suffered PONV in Group 1, and none in Group 2.

**Table 3:** Perioperative complications ^c ^Fisher’s exact test PONV, postoperative nausea and vomiting

Perioperative complications	Group 1 number (%)	Group 2 number (%)	Test-statistic (Chi-square)	p-value^c^
	Intraoperative complications			
Paresthesia	0	0	0	1
Shoulder pain	6 (13.3)	1 (2.2)	3.87	0.11
Hypotension	6 (13.3)	5 (11.1)	0.1	0.75
Bradycardia	3 (6.6)	2 (4.4)	0.21	0.73
Nausea, vomiting	1 (2.2)	0	1.01	1
Requirement of intraoperative analgesics	5 (11.1)	1 (2.2)	2.86	0.20
Requirement of additional intraoperative sedation	2 (4.4)	1 (2.2)	0.35	1
	Postoperative complications			
PONV	1 (2.2)	0	1.01	1
Urinary catheterization	1 (2.2)	0	1.01	1
Delayed recovery	1 (2.2)	1 (2.2)	0	1
Neurological sequelae	0	0	0	1

All the patients went to pass urine themselves, after two to three hours of surgery, escorted by a caretaker, except for one patient who required urinary catheterization in Group 1. Oral sips were allowed after two hours. Patients took a liquid diet six hours after the surgery. All patients were discharged after 24-36 hours, according to hospital policy. No neurological sequelae up to eight days were observed, as all the patients came to the hospital for follow-up for suture removal.

## Discussion

LC was completed successfully in all patients under TSSA. This study demonstrated that adding hypobaric bupivacaine before isobaric bupivacaine during TSSA provides better control of shoulder tip pain during LC. The hypobaric-plus-isobaric group showed considerably reduced NRS scores, better hemodynamic stability, and greater patient and surgeon satisfaction than the isobaric-alone group. In this combination, the isobaric drug takes care of the surgical anesthesia, and the hypobaric drug takes care of the shoulder pain. The baricity of LAs used has an important influence on drug spread and effect [16,17].

The incidence of shoulder pain and analgesic requirements was lower in the hypobaric + isobaric combination, with improved patient and surgeon satisfaction. The ARR for shoulder pain with the hypobaric + isobaric combination was 11.1%, yielding an NNT of nine. This indicates that approximately nine patients would need to receive the hypobaric + isobaric regimen to prevent one case of intraoperative shoulder pain. This suggests a potentially meaningful reduction in shoulder pain with the intervention, but the result is not statistically significant, due to the small sample size. The effect size for pain reduction is clinically meaningful, though satisfaction score differences are small. Adverse effects were comparable, suggesting similar safety profiles.

Although laparoscopic procedures are advantageous in terms of cosmetic scars and early recovery, a few complications, like shoulder pain, make the experience of surgery unpleasant for patients. Conducting LC under TSSA has changed this scenario. Here, the use of a low dose of bupivacaine near the target site causes minimal changes in the respiratory or cardiac system and allows rapid recovery after the procedure. The lower extremities are often spared or only minimally affected; patients do not experience problems with early mobility. This may be the reason for patient satisfaction. TSSA has also been reported for laparoscopic surgeries in pediatric patients [18].

The 2 mg of hypobaric bupivacaine in Group 2 is intended to provide targeted cephalad spread to block diaphragmatic afferents (C3-C5) for shoulder pain control, while the 9 mg of isobaric bupivacaine ensures surgical anesthesia. The additional 2 mg is a low dose, specifically chosen to achieve a segmental sensory block, without significantly increasing motor or sympathetic effects. The low concentration (0.1%) and volume (2 mL) of hypobaric bupivacaine minimize systemic absorption and toxicity risks. The total dose of bupivacaine (11 mg) in both groups remains the same and is well within safe intrathecal bupivacaine doses. Interim assessments confirmed hypobaric spread to cervical dermatomes in Group 2, supporting targeted shoulder pain control. Elbow flexion, wrist flexion, and finger movements were intact, emphasizing only sensory spread to cervical dermatomes. Literature also supports low-dose hypobaric bupivacaine (1-2 mL of 0.1%-0.15%) for targeted thoracic blocks. Nagar et al. compared TSSA with GA in two groups of 35 patients each and found it a suitable alternative to GA. They found that a combination of hypobaric and isobaric drugs can be used for LC to prevent shoulder pain. They suggested that 0.1%-0.15% and 1-2 mL hypobaric drugs, with a head-up tilt, can be used for mitigating shoulder tip pain, and 1-1.5 mL isobaric drug for LC at T10 levels [19]. Several studies reported the safety and feasibility of TSSA for LC. Chandra et al. published a large data set of 2,102 patients who received TSSA with hyperbaric bupivacaine (2.4 mL, 0.5%) and found shoulder pain in around 6%. Other hemodynamic parameters in their study were minimal and easily correctable with conventional drugs. No patient showed any neurological injury [2].

As the diaphragm is the main inspiratory muscle, which is not affected by sensory blockade, none of the patients had any respiratory issues. One case of cervical spine fixation with hypobaric drugs using TSSA emphasized that only sensory involvement at cervical roots is devoid of any respiratory compromise [20]. Intermediate cervical plexus blockade, along with TSSA, to avoid shoulder pain in laparoscopic surgeries has been reported [21,22]. However, giving two punctures - one for SA and another for an intermediate cervical plexus block - may not be accepted by awake patients.

Imbelloni found TSSA to be better than conventional LSA in 369 patients undergoing LC. They used pneumoperitoneum up to 8 mmHg and 1% lignocaine instillation at the surgical site to reduce shoulder pain; however, despite these measures, there was still an incidence of shoulder pain, although lowest in the TSSA group [23]. Vincenzi et al. conducted nine cases of LC using hypobaric-isobaric combinations and also found no shoulder pain [9]. Mahasivabhattu et al. found shoulder pain in two of 25 patients, which is around 10% [24]. They did not mention IAP. In our study, in Group 1, six (13.3%) patients had shoulder tip pain, which is quite high despite using TSSA. This may be due to the higher IAP of 10-12 mmHg used in our study. The literature suggests using a low IAP to avoid shoulder pain [25].

Precise TSSA technique effectively preserves leg movements, leading to early mobilization. All patients in our study shifted themselves with minimal support from the operating table and went for urination within two hours of surgery. Oral sips were allowed after two hours, and a soft diet was given after six hours. Here, the selection of sedation and its depth is very important. A deeper plane of sedation leads to paradoxical movement of the abdominal wall, causing difficulty for operating surgeons. Therefore, there is always a need to develop methods that can decrease shoulder pain with minimal sedation. In the past, various methods were used to decrease shoulder tip pain, such as using lower IAP, instillation of LA inside the peritoneal cavity, and the use of opioids; however, none of these methods were found to be effective in all patients [26,27]. PROSPECT guidelines recommend the use of multimodal analgesia, including regional blocks and systemic analgesics [28]. TSSA is being explored as an alternative technique in select patient groups, with limited adoption due to the limited evidence available. Its use should be restricted to controlled trials or highly selected patients until more robust evidence becomes available [29]. The use of ultrasonography in difficult spinal cases and invasive blood pressure monitoring in high-risk cases are additional safety measures to enhance the efficacy of TSSA in LC.

This study has some limitations. It was conducted in ASA I/II patients with simple surgical conditions. More data are needed to establish this technique as a routine practice. As hypobaric drugs are not commercially available, they are prepared from isobaric drugs using sterile distilled water, which may raise concerns regarding sterility and proper concentration. Anesthesiologists and patients were not blinded, which may introduce potential bias. TSSA requires specialized skills and must be performed by a senior consultant proficient in TSSA, which may not be widely available in all settings. This pilot trial suggests an association but is not definitive due to the small sample size and the limitations outlined above.

## Conclusions

In this open-label, randomized trial, the addition of a small dose of hypobaric bupivacaine to isobaric bupivacaine during TSSA was associated with a lower incidence of intraoperative shoulder pain, stable hemodynamics, and higher patient and surgeon satisfaction compared with isobaric bupivacaine alone. Approximately nine patients would need to receive the hypobaric + isobaric regimen to prevent one case of intraoperative shoulder pain. These findings are encouraging and highlight the potential of a modified technique to improve patient comfort during LC. However, the study is limited by a small sample size, partial blinding, and short follow-up for neurologic outcomes. A larger, adequately powered, double-blind, randomized trial with standardized sedation, rigorous pharmacy compounding procedures, and longer neurological follow-up is required before routine adoption.
